# Uncommon but Important: Tertiary Center Experience with Rare Cases of Breast Hamartoma

**DOI:** 10.3390/life15071076

**Published:** 2025-07-05

**Authors:** Mihaela Camelia Tîrnovanu, Bogdan Florin Toma, Elena Cojocaru, Elena Țarcă, Ștefan Dragoș Tîrnovanu, Vlad Gabriel Tîrnovanu, Cristian Mârțu, Roxana Ana Covali, Anca Irina Gradinariu, Gabriela Ghiga, Ludmila Lozneanu

**Affiliations:** 1 Department of Mother and Child Medicine, “Grigore. T. Popa” University of Medicine and Pharmacy, 700115 Iasi, Romania; mihaela.tirnovanu@umfiasi.ro (M.C.T.); gabriela.ghiga@umfiasi.ro (G.G.); 2 Department of Morphofunctional Sciences I, “Grigore. T. Popa” University of Medicine and Pharmacy, 700115 Iasi, Romania; bogdan-florin-sl-toma@d.umfiasi.ro (B.F.T.); ludmila.lozneanu@umfiasi.ro (L.L.); 3 Department of Surgery II, “Grigore. T. Popa” University of Medicine and Pharmacy, 700115 Iasi, Romania; tarca.elena@umfiasi.ro (E.Ț.); stefan-dragos.tirnovanu@d.umfiasi.ro (Ș.D.T.); 4 St. Josef Hospital, 65189 Wiesbaden, Germany; vlad.tirno@gmail.com; 5 ENT Clinic Department, “Grigore. T. Popa” University of Medicine and Pharmacy Iasi, 700115 Iasi, Romania; martu.cristian@umfiasi.ro; 6 Department of Medical Bioscience, Faculty of Bioengineering, “Grigore. T. Popa” University of Medicine and Pharmacy, 700115 Iasi, Romania; ana.covali@umfiasi.ro; 7 Department of Oral and Maxillofacial Surgery, “Grigore. T. Popa” University of Medicine and Pharmacy, 16 University Street, 700511 Iasi, Romania; anca-gradinariu@umfiasi.ro

**Keywords:** breast, hamartoma, pathology, imaging

## Abstract

**Background:** A breast hamartoma or fibroadenolipoma is a rare, benign mass consisting of disorganized mature breast tissue elements. Surgical excision is recommended if the lesion exhibits rapid progressive growth. However, incomplete excision may result in recurrence. The objective of this study is to provide comprehensive insights into the characteristics of breast hamartomas and to conduct a thorough investigation into their clinical presentation, diagnostic procedures, and management strategies. **Methods**: We report on 13 cases of breast hamartomas treated surgically between January 2018 and June 2023 at the Obstetrics and Gynecology Hospital “Cuza Vodă” in Iași. We analyzed their histological images and immunohistochemical evaluations. **Results**: The mean age of the patients was 33.35 years, ranging from 22 to 57 years. Clinically, all patients presented with a painless mass. The diagnosis was confirmed through ultrasound examination, which revealed that hamartomas appeared as well-circumscribed, oval, and heterogeneous in echotexture. The tumor sizes ranged from 1 to 17 cm, with an average size of 6.75 cm. Surgical treatment involved lumpectomy with the excision of a small portion of normal tissue surrounding the tumor. The histological variability of these tumors poses diagnostic challenges for pathologists, potentially leading to underdiagnosis. **Conclusions**: Most hamartomas exhibit characteristic features on ultrasound attributable to their fibrous, glandular, and adipose tissue composition. Accurate identification of hamartomas is crucial due to the potential for recurrence. Notably, none of the women in our study experienced recurrence during the follow-up period.

## 1. Introduction

Breast lesions are a common clinical concern, with ultrasonography serving as a vital diagnostic tool. Among the various types of breast lesions, benign tumors constitute a significant proportion. Within this category, breast hamartomas represent a distinct entity that warrants specific attention due to their unique characteristics and treatment approaches [1]. The first identification of a hamartoma as a “mastoma” was made by Pryn in 1928 [1,2]. The initial description of a hamartoma was provided by Hogeman and Ostberg in 1968, while Arrigoni first used the term “hamartoma” in 1971 [1,3,4]. The terminology currently accepted by the World Health Organization (WHO) since its inclusion in 1981 encompasses terms such as adenolipoma, chondrolipoma, and myoid hamartoma, though it does not accept terms like fibroadenolipoma or adenolipofibroma [1,5,6].

This type of tumor constitutes less than 5% of benign breast tumors, although some data indicate a prevalence as low as 0.7% [7]. It predominantly occurs in the fifth decade of life, aligning with the perimenopausal period. Etiology remains largely unknown, with ambiguity over whether it represents a malformation or a neoplasm [8].

Breast hamartoma is a benign, rare tumor comprising a disorganized mixture of mature mammary tissue elements, including glandular and stromal tissues such as fatty, fibrous, adenomatous, and pseudoencapsulated lobular components, often presenting as palpable masses or incidental findings on imaging studies. These elements are unsystematic, with lobules varying in size and shape, often fusing or being disorganized. Myoid hamartomas, a subtype in which smooth muscle is prominent, can also be observed [9,10]. Smooth muscle fibers within the tumor can be highlighted through immunohistochemical examination (IHC) using desmin or actin [1]. Estrogen receptor (ER) and progesterone receptor (PR) expression in hamartomas is similar to that in normal breast tissue epithelium [11]. Additionally, IHC shows CD34 distribution in intralobular, perilobular, and interlobular regions [12], with p63, CK5/6, calponin, and myosin being positive in myoepithelial cells [13].

Differential diagnosis must be performed with fibroadenoma, which has more architectural organization and less cellular stroma with the absence of lobules and fat; breast lipoma composed of lobulated mature adipose tissue, with minimal connective tissue stroma; and Phyllodes tumor characterized by cleft-like epithelium-lined spaces and more cellular stroma [14].

The objective of this study is to provide comprehensive insights into the characteristics of breast hamartomas and to conduct a thorough investigation into the clinical presentation, diagnostic procedures, and management strategies for breast hamartomas in a gynecology department in Romania. By focusing on this specific benign condition, we aim to elucidate the nuances of its clinical features and the challenges associated with its diagnosis and treatment. We analyzed histological images and immunohistochemical evaluations. It is crucial to raise awareness of this underrecognized benign condition, as it can clinically resemble other breast tumors, both benign and malignant.

## 2. Materials and Methods

A total number of 543 patients with breast lesions were examined using ultrasonography between January 2018 and June 2023 at the “Cuza Vodă” Obstetrics and Gynecology Hospital in Iași, Romania. The number of patients with benign tumors on ultrasound was 320, representing 60% of the total patients. All cases of breast hamartoma that were surgically treated were selected from this group. Our analysis is not a comprehensive evaluation of all benign breast tumors, but rather a focused investigation on breast hamartomas. The inclusion criteria were as follows: female patients with a confirmed diagnosis of breast hamartoma through histopathological examination; cases fully documented with available clinical, imaging, and histopathological data in the medical record; post-diagnosis monitoring of at least 6 months to observe patient evolution. The exclusion criteria were as follows: patients with ambiguous diagnoses or without histopathological confirmation of hamartoma; the presence of concurrent malignant breast lesions, which may complicate the interpretation of data specific to hamartoma; incomplete data in medical records, such as missing imaging studies necessary for the initial assessment; patients who refused long-term monitoring or did not follow clinical recommendations, thus affecting the objective evaluation of lesion progression. The final count was 13 patients.

Breast ultrasound was performed on all patients preoperatively. US examinations were conducted by gynecologists with experience in breast imaging using the GE Voluson E8 Expert ultrasound system with an L12-5 linear array probe. All patients were positioned in lateral decubitus with the ipsilateral arm raised and the hand placed under the head. A radial exploration approach was employed to ensure that no lesions were overlooked. Upon identification, each lesion was re-evaluated by measuring two dimensions. The following details about the lesion were documented: location, largest diameter and overall size, margins, internal echogenicity, and blood flow signal.

In our cases, an excisional biopsy with intraoperative frozen section analysis was chosen. This decision was based on the ultrasound criteria, which did not suggest malignancy in any of the cases. Therefore, the direct excisional biopsy approach allowed for accurate diagnosis while minimizing the risk of misdiagnosis. The surgical approach for breast hamartoma consisted of a lumpectomy, with a margin of healthy tissue surrounding the lesion.

The follow-up for patients involved an ultrasound evaluation at 2 months postoperatively, followed by assessments at 6 months and then annually thereafter. Each patient also underwent clinical evaluations during these follow-up visits. The absence of recurrence based on these clinical and ultrasound evaluations, confirming no signs of new lesions or tumor growth throughout the follow-up period was defined.

This study was conducted according to the guidelines of the Declaration of Helsinki and approved by the Ethics Committee of ′′Cuza Voda′′ Clinical Hospital of Obstetrics and Gynecology, Iasi, Romania (no. 10029/05.08.2024). Written informed consent has been obtained from all the patients to publish this paper.

## 3. Results

The clinicopathologic features of the patients included in the study are outlined in detail in Table 1.

The patients’ average age was 33.35 ± 4.5 years, ranging from 16 to 57 years, with five patients aged between 20 and 25 years. Clinically, the presentation was typically a well-defined, mobile, and painless tumor. Patients reported the onset of the tumor occurring between 1 month and 1 year before diagnosis. The exact etiology of hamartoma growth remains unknown, and cases are generally sporadic.

Out of the thirteen cases, seven tumors were located in the right breast, and eight were found in the external superolateral quadrant (ESQ). Even though the fact that hamartomas do not have a specific localization within the breast, in eight out of the thirteen women, the tumor was situated in the external superolateral quadrant.

**Figure 1:**
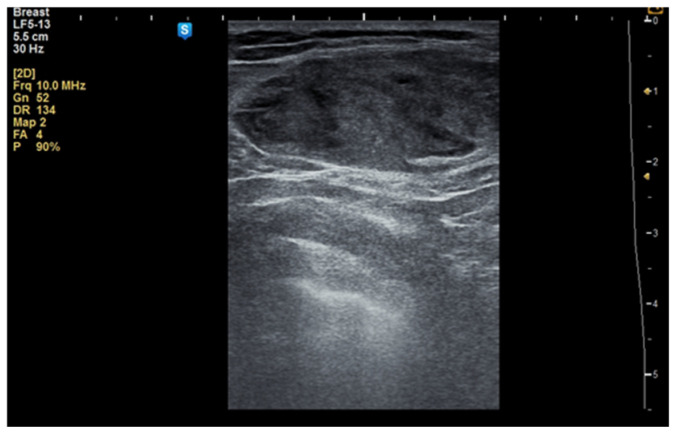
Ultrasound image of a 34-year-old woman with a right breast hamartoma of 65/35 mm, with heterogeneous hypoechoic structure, located in the upper inner quadrant.

**Figure 2:**
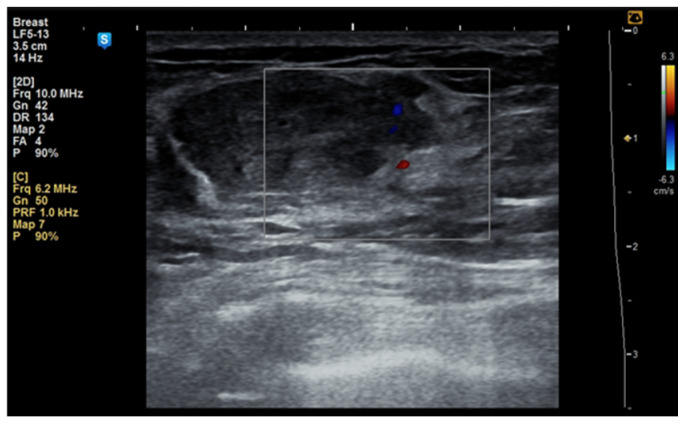
Ultrasound image of a 53-year-old woman with a right breast hamartoma with reduced vascularity, located in the lower inner quadrant.

Although the literature identifies mammography as the primary diagnostic method for breast hamartomas, in our study, the diagnosis was established via ultrasound examination. This preference is due to the younger age of many patients, with only three being over 50 years old. On ultrasound, hamartomas typically appear as oval, well-circumscribed masses with heterogeneous hypoechoic or isoechoic echostructures and reduced or absent vascularity (Figure 1 and Figure 2). In our study, the concordance between ultrasound and histological size was 84.61%. Preoperative ultrasound diagnosis of hamartoma was established in only five patients, representing 38.46% of the cases.

**Figure 3:**
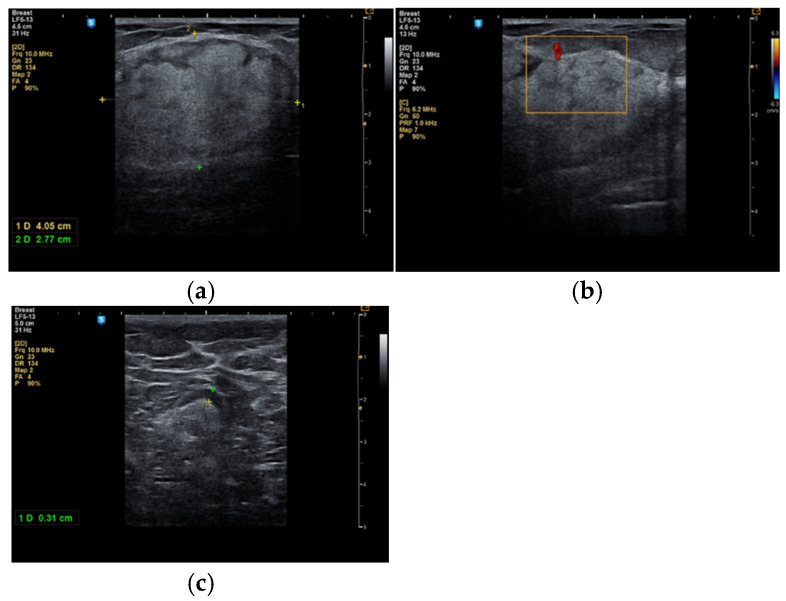
A 53-year-old woman with a tumor in the right upper outer mammary quadrant (**a**) with predominant hyperechoic structure; (**b**) with a peripheral vessel; (**c**) with benign axillary adenopathy.

In some cases, the diagnosis was initially suspected to be a malignant tumor (Figure 3a–c).

**Figure 4:**
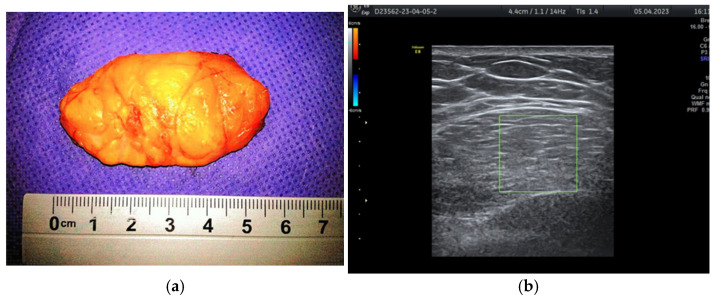
Breast lipoma in a 52-year-old woman (**a**) macroscopic pathological aspect; (**b**) ultrasound exam—hypoechoic aspect with thin striations.

During the study period, a case initially suspected to be a hamartoma on MRI, with peripheral vessel involvement and benign axillary adenopathy, was encountered. However, an ultrasound examination established the diagnosis of a 5 × 3 cm lipoma in a 52-year-old woman, despite the tumor’s rapid growth over the course of one year (Figure 4a,b). The lipoma consisted solely of homogeneous adipose tissue, appearing hypoechoic on the ultrasound exam, with few thin striations. This case is particularly significant as it highlights the diagnostic challenges associated with differentiating between breast hamartomas and lipomas, especially in instances where rapid tumor growth is observed over one year.

**Figure 5:**
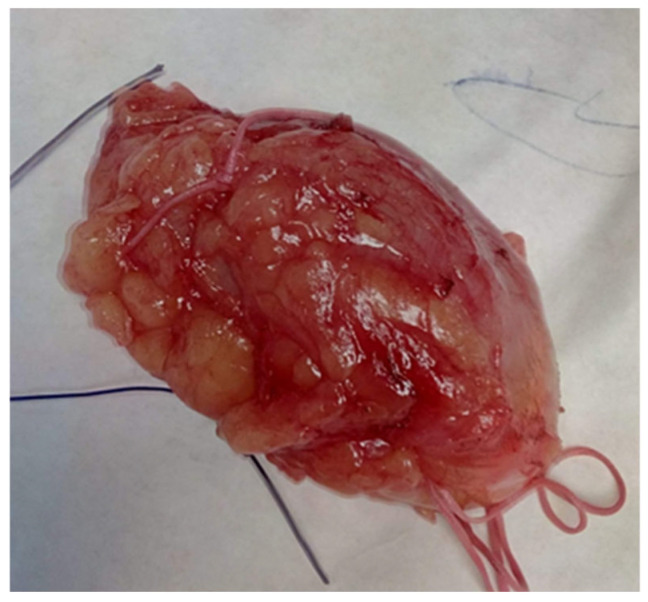
Hamartoma excised with surrounding normal breast tissue, marked for frozen section.

Surgical treatment for cases of breast hamartoma involved lumpectomy with a margin of normal tissue surrounding the tumor (Figure 5) to minimize the risk of recurrence. In all cases, frozen section analysis was performed.

Regarding the macroscopic appearance of the tumors, their sizes ranged from 1 cm to 17 cm, with an average of 6.75 cm. Hamartomas in adolescent girls can grow to considerable sizes (>10 cm) and may resemble a giant juvenile fibroadenoma. For instance, a single case of a 16-year-old with a 5 cm tumor was diagnosed.

**Figure 6:**
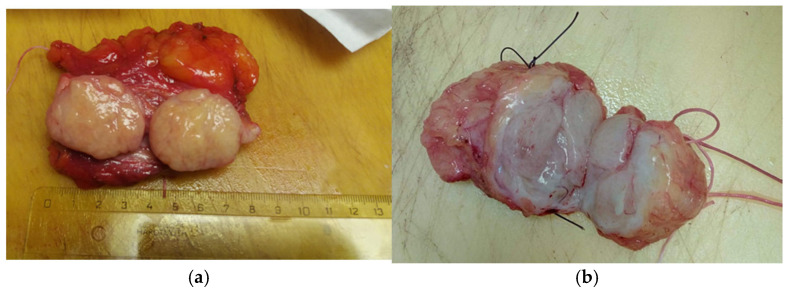
The hamartoma section (**a**) yellow or (**b**) gray.

Typically, the tumor is enclosed in fibrous tissue that delineates it from the surrounding breast tissue. Upon sectioning, the hamartoma presents as a fleshy mass with a rubber-like consistency and exhibits yellow or gray coloration (Figure 6a,b).

**Figure 7:**
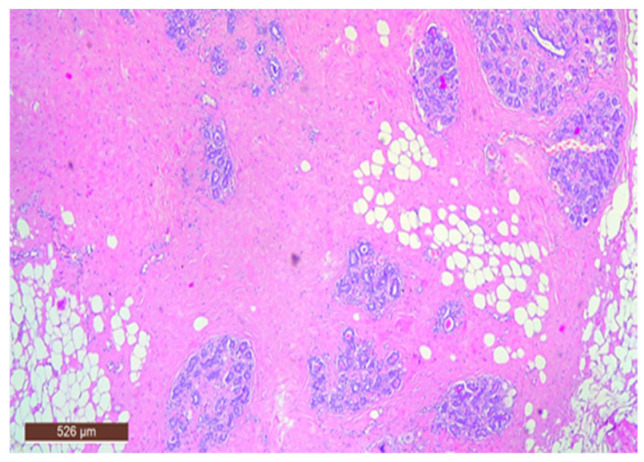
Hamartoma—histological structure (HE ×40).

**Figure 8:**
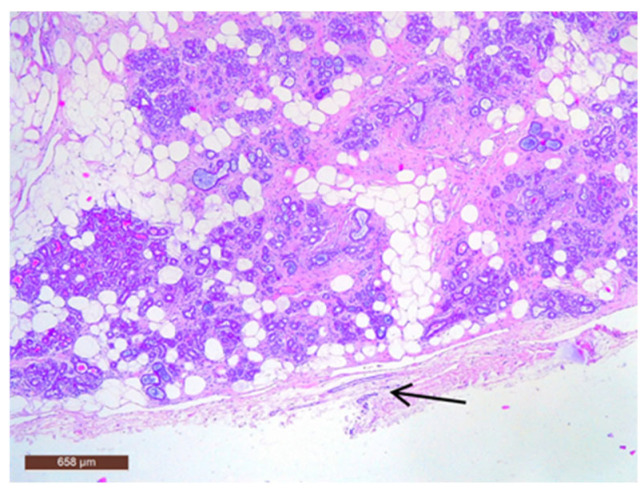
Hamartoma with fine connective tissue capsule (arrow) (HE ×40).

**Figure 9:**
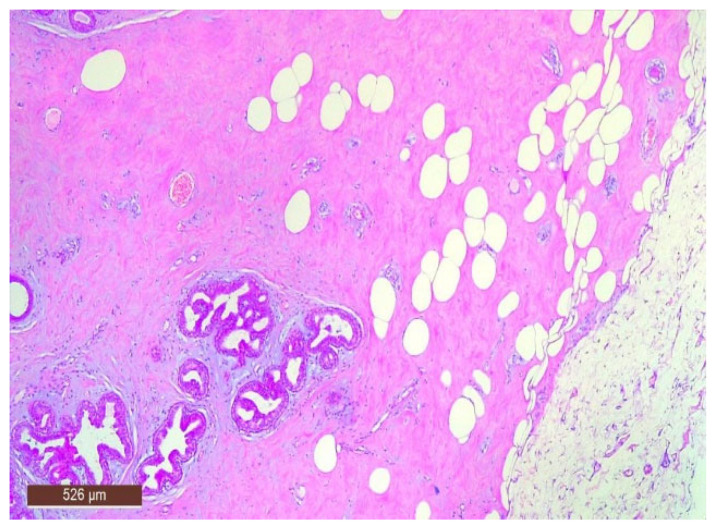
Ducts with apocrine metaplasia inside a hamartoma (HE ×40).

On microscopic examination, a hamartoma may exhibit three predominant components: glandular, fibrous and adipose (Figure 7). Epithelial components such as ducts and lobules are generally normal, featuring a single layer of epithelium overlying the myoepithelial cells. The fibrous component was composed of connective tissue stroma with a delicate connective tissue capsule (Figure 8). Additionally, the ducts within the hamartoma may display apocrine metaplasia (Figure 9).

**Figure 10:**
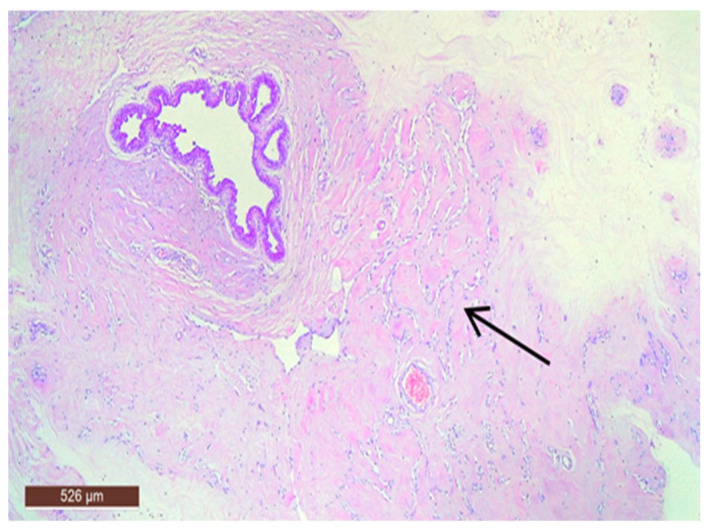
Hamartoma with pseudoangiomatous stromal hyperplasia (HE ×40).

The stromal component of the lesion is frequently hyalinized and poorly defined, extending around and infiltrating lobular structures, and may exhibit characteristics of pseudoangiomatous stromal hyperplasia (PASH) (Figure 10). Epithelial hyperplasia is uncommon, with only one case of atypical hyperplasia observed in our study.

**Figure 11:**
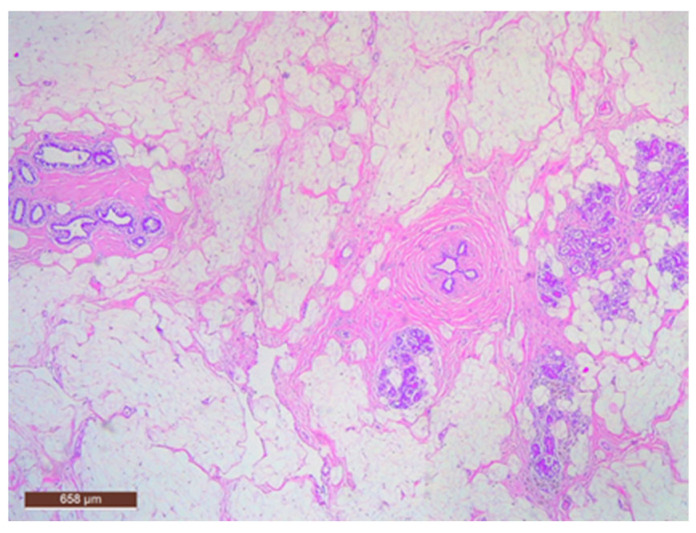
Hamartoma with fat cells interspersed within the lesion (HE ×40).

Adipose tissue in the hamartoma may be prominently proliferative (Figure 11), which can complicate the differential diagnosis with a lipoma on ultrasound. Conversely, the adipose component can also be nearly absent.

**Figure 12:**
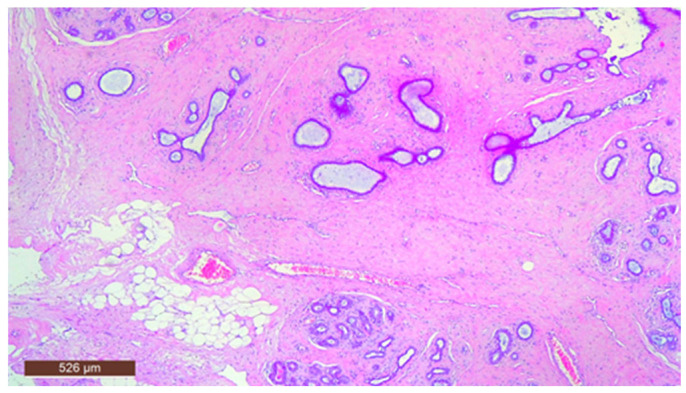
Hamartoma with prominent fibrous tissue (HE ×40).

In some instances, fibrous tissue may predominate (Figure 12). Additionally, three cases in our study were associated with chronic inflammation. In the series of 13 cases, no cases with associated malignancy were observed.

In our study, the immunohistochemical examination (IHC) was used to support the diagnosis of breast hamartoma, helping to confirm its benign nature and to differentiate it from other breast lesions with overlapping histological characteristics.

**Figure 13:**
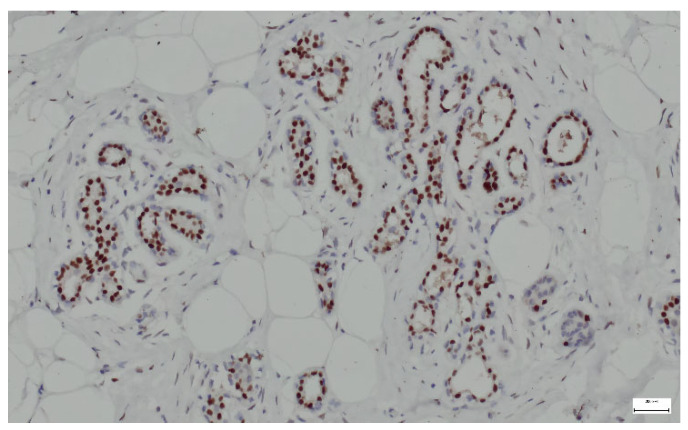
ER lining the ducts and lobules in breast hamartoma (anti-ER, ×10).

Breast hamartomas often express estrogen receptors (ER), that can be detected in the epithelial cells lining the ducts and lobules within the hamartoma. ER expression was found in all tissue samples (Figure 13).

**Figure 14:**
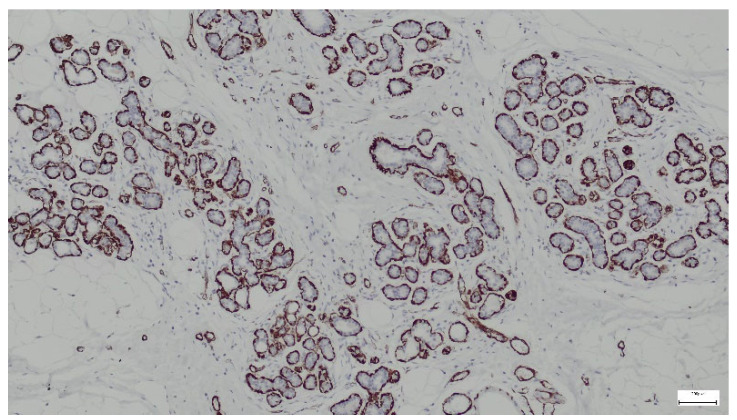
SMA highlights myoepithelial cells surrounding ducts and lobules in the breast hamartoma (anti-SMA ×10).

Smooth muscle actin (SMA) is a type of actin found predominantly in smooth muscle cells but can also be expressed in myoepithelial cells (surrounding glandular structures), myofibroblasts (in the stromal component), and pericytes (in blood vessel walls). In our study, we used the immunohistochemical marker SMA to identify these cell types in hamartoma sections. All samples analyzed in our study showed positive expression for SMA (Figure 14).

**Figure 15:**
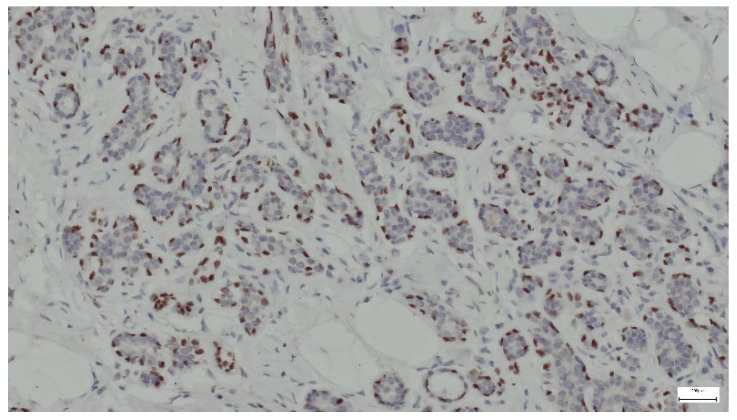
p63 expression in ducts and lobules lined by epithelial and myoepithelial cells (anti-p63 ×10).

p63 is a protein that serves as a marker for basal cells in various tissues, including the breast. In the context of breast hamartoma, p63 expression can help differentiate between benign and malignant lesions and may provide insights into the myoepithelial cell population within the tumor. In all cases, p63 was strongly expressed in the nuclei of myoepithelial cells surrounding the ducts and lobules (Figure 15).

## 4. Discussion

The distinction between various types of benign breast tumors is crucial for appropriate management and treatment. Breast hamartomas are a unique and often underrecognized entity that can pose diagnostic dilemmas due to their clinical presentation, which may closely resemble other breast tumors, including both benign and malignant forms. This overlap in presentation can lead to misdiagnosis and inappropriate management. According to the literature, breast hamartomas are typically present in the fifth decade of life; however, in our study, the ages ranged from 16 to 57 years [15]. The effectiveness of imaging modalities in detecting breast hamartomas reported in the literature was 30% for mammography, 18% for ultrasound, and 68% for MRI [16]. In our study, the diagnostic success rate for breast hamartoma by ultrasound was 38.46%.

Although breast hamartomas lack distinctive histological features, a thorough evaluation is essential for accurate diagnosis. A review of 25 cases of breast hamartoma highlights the limitations of various diagnostic methods, including imaging, fine needle aspiration cytology (FNAC), and fine needle core biopsy (FNCB). The findings suggest that these tumors are challenging to diagnose solely based on histology and correlating clinical and radiological data is crucial to avoid underdiagnosis [17,18]. In our study, fine needle aspiration biopsy (FNAB) and FNCB were not performed because the ultrasound characteristics suggested a benign lesion.

The decision not to perform FNAB or a core biopsy was based on the benign appearance (BI-RADS 2 or 3) of the lesions from the ultrasound [18]. No case in our cohort was classified as BI-RADS 4, 5, or 6. As per current guidelines, BI-RADS 3 lesions carry a malignancy risk of <2%, and biopsy is not mandatory unless there are personal or family risk factors for breast cancer [19]. In the one case rated BI-RADS 3, the patient chose surgical excision to avoid the psychological stress of prolonged imaging surveillance. Moreover, FNAB and core needle biopsy are not always reliable in diagnosing hamartomas, as they may not capture the characteristic mixed architecture. All patients in our study underwent intraoperative frozen section examination to rule out in situ or invasive carcinoma.

The molecular data on breast hamartomas are limited. Molecular or cytogenetic description indicates translocation in HMGA2 and germline mutations of the tumor suppressor gene PTEN [20]. It seems that hamartoma stromal cells expressed HMGA2, ER, and PR in 79%, 66%, and 76.3% of cases, respectively, compared to 7.7%, 23%, and 19% in normal breast tissue, respectively (*p*  <  0.0001; *p*  =  0.0005; *p*  <  0.0001) [21,22].

This type of breast tumor can also be found in patients with certain syndromes such as Cowden disease or Peutz–Jeghers syndrome. Cowden disease, which involves mutations in the tumor suppressor gene PTEN, is associated with a spectrum of breast pathologies: approximately 22% of women with Cowden disease develop breast carcinoma, and over 66% have fibrocystic disease [23]. While mammary hamartomas occur in only 0.1–0.7% of the general population, they are more commonly found in individuals with Cowden disease [24]. While benign breast lesions such as fibrocystic disease or hamartomas do not meet the major diagnostic criteria for Cowden disease, their presence in the appropriate clinical context can be a significant factor suggesting this diagnosis. The diagnosis of Cowden disease involves a combination of clinical and genetic criteria, and finding benign breast lesions, especially when accompanied by other characteristic clinical features, may help guide clinicians toward this diagnosis. Peutz–Jeghers syndrome is another genetic condition characterized by the presence of hamartomas in multiple locations, including the mouth, colon, breast, neck, testicles, and pancreas. Individuals with Peutz–Jeghers syndrome also have an increased risk of developing cancers in various organs, making early detection and monitoring crucial [25]. Although the literature describes associations between breast hamartomas and certain genetic syndromes (e.g., Cowden syndrome) [26], no genetic investigations were performed on the patients included in our study. Breast, ovarian, and cervical tumors are associated not only with genetic syndromes but also with some unfavorable socio-economic conditions, which overall decrease both fertility rates and life expectancy in some countries [27,28].

Clinically, breast hamartomas are often present as a prominent palpable tumor or obvious breast asymmetry, with sizes varying from 1 to 15 cm, occasionally presenting as giant forms [15]. Consistent with existing data, in our study, tumor sizes ranged from 1 cm to 17 cm. Sometimes, clinical diagnosis is difficult due to the small size and consistency of the lesion similar to that of the mammary gland. Growth is typically slow, often spanning several years before reaching a noticeable size. The tumors have no specific localization within the breast [15]. Mammary hamartomas primarily occur in the female breast. However, they have also been reported in the male mammary gland, in ectopic breast tissue, and in patients with associated breast carcinomas [23].

In mammographic screenings, the reported incidence of breast hamartomas is 8% [29]. The classic mammographic appearance of a breast hamartoma is highly characteristic and allows for a definitive diagnosis: the lesion is well-circumscribed, containing both fat and breast tissue, and is surrounded by a thin radiopaque capsule, which becomes visible when fat is present on both sides. In addition, the appearance resembles a “cut salami” on an X-ray or a “breast within a breast”. Although many of these lesions lack a true fibrous capsule, a thin radiopaque pseudocapsule typically surrounds at least part of the mass [30].

Hamartomas are predominantly composed of fibrous tissue and pose challenges in diagnosis via mammography as they often resemble fibroadenomas or carcinomas due to minimal or absent intratumoral fat. According to Masciadri et al., ultrasound examination shows a vascular score of 50% score 1, 36.66% score 0, and 13.34% score 2 [31]. Elastography reveals a color pattern with predominant dark blue and light blue areas, indicating reduced elasticity compared to surrounding tissue, likely due to higher fibrous content [16,32].

While MRI is typically not the primary diagnostic tool for suspected hamartomas, it confirms intratumoral fat presence. Also, hamartomas appear smooth, intense, and heterogeneous on MRI, facilitating distinction from normal breast tissue and detailed assessment of margins and structure. MRI becomes necessary when radiological and clinical findings conflict. Hamartomas typically show denser characteristics than normal breast tissue, with parenchymal elements occasionally enhancing after gadolinium administration, albeit less intensely than malignant lesions [8]. The absence of mammography and MRI may reduce comparability with other studies; however, most of our patients were young women with dense breast tissue, where mammography is often of limited diagnostic value. In such cases, ultrasound remains the preferred initial imaging modality. Additionally, MRI is not routinely used for the assessment of typical hamartomas, especially in the absence of suspicious features. As highlighted by Cucci et al. (2015) and Rosen & Siegelman (2005), MRI findings in hamartomas are non-specific and are often reserved for atypical or complex cases [33,34]. Another study by Sevin et al. that analyzed 27 cases of breast hamartoma evaluated the patients preoperatively only by ultrasound [35].

Breast ultrasound was performed on all our patients preoperatively. In cases of breast hamartoma, neither preoperative mammography nor MRI were used. Breast MRI is not routinely employed in our practice due to the high cost of this investigation in our country.

Core biopsy plays a limited role in definitive diagnosis and necessitates clinical and radiological correlation to prevent underdiagnosis. Surgical excision is recommended for rapidly growing lesions. Despite their benign nature, hamartomas can coexist with malignancy in 0.1% of cases due to their epithelial components [36]. A literature review reports 15 cases of carcinoma associated with hamartomas, often identified through suspicious mammographic or ultrasound features (e.g., microcalcifications, spiculated opacities, irregular hypoechoic lesions) [37]. In some instances, malignancy is unexpectedly discovered during histological examination post-lumpectomy. Therefore, recognizing suspicious ultrasound and mammographic features in hamartomas is crucial. Incomplete excision may lead to hamartoma recurrence [38].

Histological diagnosis of hamartomas can be challenging due to their similarity to normal breast tissue, and diagnosis is often confirmed through appropriate imaging findings. When imaging raises suspicion, the pathologist will examine the specimen with this context in mind [8]. The variability in histological features can complicate diagnosis and may contribute to underdiagnosis; for instance, two cases were initially diagnosed as breast fibroadenomas and one case as fibrocystic mastosis based on frozen section analysis. Although not exclusive to hamartomas, the presence of interlobular fibrosis and fat within the stromal component are crucial features to identify when suspicion arises.

The proportion of stroma, adipose tissue, and glandular tissue in breast hamartoma does not demonstrate predictive value regarding the clinical behavior or prognosis of the lesion. Despite variations in the composition of these tissue types, they do not correlate with significant outcomes such as growth patterns, recurrence rates, or the likelihood of associated complications. This lack of correlation suggests that other factors may be more critical in determining the clinical significance of breast hamartomas, emphasizing the need for a comprehensive approach in their assessment and management, as well as the need for additional investigations on the entire female reproductive system, as associations with ovarian tumors are possible [8,39].

In addition to histological evaluation, breast hamartoma can be confirmed by IHC, where glandular components that contain normal breast ducts and lobules are highlighted by ER, SMA, and p63. Immunostaining aids in the differentiation of breast hamartomas from malignant lesions and contributes to a detailed characterization of these benign entities. ER expression in breast hamartomas can indicate hormone responsiveness, similar to other benign and malignant breast lesions. ER can also be expressed in the stromal component of the hamartoma, though this is less common. Smooth muscle actin (SMA) staining in breast hamartomas can help identify the presence and distribution of myoepithelial cells around glandular structures. SMA can also stain myofibroblasts in the fibrous stroma of breast hamartomas. The staining can highlight pericytes and smooth muscle cells in the walls of blood vessels within the hamartoma [40]. p63 is a member of the p53 family of transcription factors and is involved in the development and maintenance of epithelial tissues. It is commonly used as a marker for myoepithelial cells in the breast and other tissues. The detection of a continuous layer of p63-positive myoepithelial cells around ducts and lobules supports the diagnosis of a benign lesion [40].

Differential histological diagnosis should consider fibroadenoma or phyllodes tumors [8]. Fibroadenomas typically exhibit more defined architectural organization, with a proliferation of specialized stroma around lobules and the absence of lobules and fat. In contrast, phyllodes tumors are characterized by spaces lined with epithelium-like clefts and a more cellular stroma.

The limitations of the study include the small number of cases due to the rarity of breast hamartoma, incomplete or inconsistent medical data, variability in patient monitoring, the risk of ambiguous diagnoses, and potential selection bias. These constraints may limit the generalizability of the results and the accuracy of interpretations.

## 5. Conclusions

Most hamartomas exhibit a characteristic ultrasound appearance, correlating with their fibrous, glandular, and adipose tissue composition. Ultrasound provides detailed insights into the boundaries, nature, content, mobility, and homogeneity of the breast lesion, which mammography cannot offer comprehensively. However, achieving a definitive diagnosis using a single imaging modality remains challenging. Hamartomas lack distinct histological features, making pathological diagnosis difficult. The presence of adipose tissue within a breast tumor can guide the pathologist toward a diagnosis of hamartoma. Accurate identification of hamartomas is crucial due to potential recurrence issues. In our study, no recurrences were observed during the follow-up period. Currently, surgical excision of breast hamartomas is considered to be curative. This study seeks to delineate the surgical management of breast hamartomas, thereby contributing to the existing literature on benign breast tumors while emphasizing the need for targeted research in this area.

## Figures and Tables

**Table 1:** The clinicopathologic characteristics of the 13 patients.

Number of Patients	No. 13
Age	16–57 years (mean 33.35 ± 4.5)
Tumor size (cm)	1–17 cm (mean 6.75)
Onset of the tumor	1 month → 1 year
Location of the tumor	7 (right breast) 4 (left breast)
Additional lesions	
Ductal epithelial atypical hyperplasia Pseudoangiomatous hyperplasia Myoid hamartoma	1 (7.69%) 2 (15.38%) 1 (7.69%)
IHC	
SMA ER p63	positive positive positive
Preoperative evaluation	
Ultrasonography	13 (100%)
Surgery	
Lumpectomy with frozen sections	13 (100%)

## Data Availability

The original contributions presented in the study are included in the article, further inquiries can be directed to the corresponding authors.
